# Chrysin sensitizes glioblastoma cells and spheroids to temozolomide treatment by reducing EMT and stemness phenotypes, as well as targeting multidrug resistance proteins

**DOI:** 10.3389/fphar.2025.1643186

**Published:** 2025-09-02

**Authors:** Yunus Aksüt, Aslıhan Şengelen, Dudu Melek Gürsoy, İrem Öğütcü, Özge Kuvet, Murat Pekmez

**Affiliations:** ^1^ Department of Molecular Biology and Genetics, Faculty of Science, Istanbul University, Istanbul, Türkiye; ^2^ Department of Molecular Biology and Genetics, Basic Medical Sciences, School of Medicine, Koç University, Istanbul, Türkiye; ^3^ Department of Molecular Biology and Genetics, Institute of Graduate Studies in Sciences, Istanbul University, Istanbul, Türkiye

**Keywords:** glioblastoma cells and spheroids, temozolomide (TMZ), chrysin (CHR), drug synergism, multidrug resistance proteins, cellular response

## Abstract

**Background:**

Glioblastoma (GB, grade-IV astrocytoma) is a highly aggressive brain tumor often resistant to treatment with temozolomide (TMZ) due to multidrug resistance (MDR). Researchers are investigating natural compounds, such as chrysin (CHR), with anti-cancer properties; however, its ability to overcome drug resistance remains unclear. This study aimed to evaluate the possible synergistic effects of CHR and TMZ on glioblastoma cells in 2D- and 3D-culture models.

**Methods:**

Based on cytotoxicity (MTT test) and synergism analysis, U-87MG cells were treated with CHR (25 μM) and TMZ (250 μM), individually or combined, for 48 hours. Clonogenicity, migration, and invasion were assessed. Fluorescence staining was used to assess MtMP collapse, ER stress, autophagy, apoptosis, and target protein localization. Protein level alterations were measured using Western blotting, and network pharmacology was used to identify shared molecular targets. Antitumor effects were also assessed in 3D-tumor spheroids (mimics *in vivo* tumors), through viability and growth analyses.

**Results:**

The combined treatment was more effective in reducing cell proliferation than either agent alone, in a dose- and time-dependent manner. CHR increased TMZ cytotoxicity by promoting mitochondrial dysfunction, ER stress, autophagy, and apoptosis, and further decreased motility, clonogenicity, EMT status, and stem-like traits. Co-treatment also suppressed the TMZ-induced upregulation and nuclear translocation of P-glycoprotein (identified as a key CHR target through network pharmacology analysis) and NF-κB-p65, as well as reduced the expression of stress proteins (Hsp60, Hsp70, Hsp90) and MRP1. In 3D spheroid models, co-treatments significantly impaired growth and viability.

**Conclusion:**

These findings suggest that CHR may be a promising adjuvant to TMZ therapy, providing novel insights into overcoming chemoresistance in GB treatment.

## 1 Introduction

Glioblastoma (GB, grade-IV astrocytoma) is a highly aggressive and common malignant brain tumor in adults. It is characterized by rapid progression, diffuse infiltration, and resistance to conventional therapies. Despite advancements in surgical resection, radiotherapy, and chemotherapy, the prognosis for GB patients remains unfavorable, with a median survival rate of approximately 12–15 months following diagnosis (Grochans et al., 2022). Temozolomide (TMZ) is the primary drug for treating GB. However, its effectiveness is often hindered by chemoresistance due to mechanisms such as increased DNA repair capability, alterations in apoptotic pathways, enhanced autophagy, and survival signaling. These resistance mechanisms not only reduce the cytotoxic effects of TMZ but also contribute to tumor recurrence and poor clinical outcomes (Singh et al., 2021). Identifying agents that enhance TMZ sensitivity or overcome resistance is crucial. Despite improvements with chemotherapeutic TMZ and non-invasive tumor-treating fields, GB’s aggressiveness necessitates additional treatment strategies to improve patient survival rates and quality of life (Pouyan et al., 2025).

Given the longstanding issues of side effects and chemotherapy resistance associated with TMZ, research has increasingly focused on supportive natural therapies. Chrysin (5,7-dihydroxyflavone, CHR), a natural flavonoid found in honey, propolis, and various plant extracts, has garnered attention for its pharmacological properties, including anti-inflammatory, antioxidant, and anticancer effects (Kasala et al., 2015; Mani and Natesan, 2018). Emerging evidence indicates that CHR can influence molecular pathways involved in cell proliferation, apoptosis, and drug resistance. Its potential therapeutic anticancer effects have been reported in many cancers, including bladder (Xu et al., 2018; Lima et al., 2020), brain (Weng et al., 2005; Wang et al., 2018; Mahdi et al., 2023), breast (Lirdprapamongkol et al., 2013; Yang et al., 2014), cervix/ovary (Lim et al., 2018; Raina et al., 2021), colon (Lin et al., 2018), gastric (Xia et al., 2015; Zhong et al., 2020; Lee et al., 2021), liver (Xu et al., 2017), lung (Brechbuhl et al., 2012; Tang et al., 2024), pancreas (Zhou et al., 2021), and prostate (Ryu et al., 2017). Notably, CHR has been investigated for its potential to enhance therapeutic effects in cancer cells that exhibit resistance to chemotherapeutic agents such as cisplatin, doxorubicin, and 5-fluorouracil (Raina et al., 2024). However, its role in modulating TMZ resistance in glioma cells is not well understood. Research conducted by Liao et al. (2014) has demonstrated that CHR administration increases the sensitivity of GBM8901 glioblastoma cells to TMZ; this report was limited to 2D models and did not investigate the underlying molecular mechanisms. Our study systematically assesses the CHR–TMZ combination in 2D and 3D U-87MG glioblastoma models, examining apoptosis, autophagy, mitochondrial membrane potential (MtMP), endoplasmic reticulum (ER) stress, epithelial-mesenchymal transition (EMT), cancer stem cell (CSC) markers, multidrug resistance (MDR) proteins, and nuclear factor-kappaB (NF-κB)/P-glycoprotein (P-gp/ABCB1) signaling using experimental and bioinformatic methods. These findings offer a clearer mechanistic and translational perspective on CHR–TMZ synergy in glioblastoma.

This study sought to determine whether CHR can enhance the cytotoxic effects of TMZ and to investigate the underlying molecular mechanisms involved. We examined the influence of CHR on TMZ resistance in the U-87MG glioblastoma cell line, which is a widely utilized *in vitro* model for studying GB biology and treatment responses. Our findings indicated that treatments with the CER and TMZ decrease the viability of glioma cells in a manner dependent on both dosage and duration. CHR showed a synergistic cytotoxic effect with TMZ; CHR + TMZ treatments exhibited a significant anticancer impact compared to single-agent applications. Combined treatment attenuated cell proliferation, motility, clonogenic survival, and the EMT/stem-like state and led to the loss of MtMP, induction of ER stress, autophagy, and apoptosis. Notably, pre-treating with wortmannin (an autophagy inhibitor) reversed CHR + TMZ cytotoxicity and reduced LC3A-II and cleaved caspase-3 levels. Remarkably, despite increased levels of stress/heat shock proteins (Hsp60, Hsp70, and Hsp90), a key cellular transcription factor NF-κB-p65, and MDR proteins P-gp and MDR-associated protein 1 (MRP1/ABCC1) resulting from TMZ treatment, CHR co-treatment prevented the increased protein expression profile in glioma cells. Additionally, the increased nuclear localization of P-gp and NF-κB after TMZ treatment decreased with CHR application. Remarkably, CHR + TMZ treatments significantly reduced the viability and growth of cancer stem cell-like 3D-spheroids (an *in vitro* system that mimics *in vivo* tumors). This study is the first to report on the potential anticancer mechanism of CHR plus TMZ combined treatment in both 2D-monolayer and 3D-spheroid models. Moreover, the network pharmacology approach revealed 20 overlapping target genes between CHR, TMZ, and glioma, linking them to oncogenic signaling pathways. CHR was also uniquely associated with 60 glioma-related genes, including drug resistance protein P-gp, as confirmed by immunoblotting and immunofluorescence labeling. Obtained results suggest that CHR enhances the efficacy of the chemotherapeutic drug TMZ and may provide new insights into the potential application of CHR as an adjuvant therapeutic agent in the management of gliomas. Therefore, after further *in vitro* and *in vivo* studies, using CHR plus TMZ might be a promising approach to treating GB cancers.

## 2 Materials and methods

### 2.1 2D-cell culture and conditions

The study was conducted with U-87MG human glioblastoma cells obtained from Istanbul University Cell Culture Collections. Cells were cultured in DMEM supplemented with 10% FBS, 1% antibiotic-antimycotic solution (100 U/mL penicillin, 100 μg/mL streptomycin, 0.25 μg/mL amphotericin-B), and 1% NEAA in a humidified atmosphere containing 5% CO_2_ at 37 °C. The experiments were conducted with logarithmically growing cells (passages 3–10) as well as cultures initiated with cells from different passages. Standard culture reagents were from Gibco (Carlsbad-USA).

### 2.2 Cytotoxicity assay, drug synergism analysis, and treatments

MTT assay was performed to determine the cytotoxic effects and IC_50_ (half maximal inhibitory concentration) values of temozolomide (TMZ, #T2577, Sigma/St. Louis-USA) and chrysin (CHR, #95082, Sigma/St. Louis-USA) as described previously (Şengelen and Önay-Uçar, 2024). Exponentially growing-cells were plated into 96-well microplates (2 × 10^4^ cells/well). Therapeutic reagents were dissolved in DMSO, and different doses of CHR (concentration range of 0–100 μM) and TMZ (concentration range of 0–1000 μM) were applied for 24-h, 48-h, and 72-h. Afterwards, MTT solution (5 mg/mL in D-PBS) was added for 4-h, DMSO was added to dissolve the formazan crystals, and absorbance at 540 nm was measured.

To evaluate the *in vitro* combination effects of CHR (10, 25, 50, 75, and 100 µM) and TMZ (100, 250, 500, 750, and 1000 µM), synergism analysis was performed using the percentage inhibitory effect of either alone or in combination with both agents on cell viability (as determined by MTT test for 48-h). The SynergyFinder software v3.0 (https://synergyfinder.fimm.fi/) was utilized to assess the synergistic effect and calculate the synergy score (SS) (Ianevski et al., 2022). Synergism analysis was conducted using the Bliss, Loewe, ZIP, and HSA synergy score models. To validate the drug synergistic interaction, doses with SS values greater than 10 in the drug-drug matrix were selected. Additionally, the combination index (CI) by the Chou-Talalay method (Chou and Talalay, 1984) was utilized to quantify the *in vitro* drug combination effects using Compusyn software (v1.0, Compusyn Inc., Paramus/USA). CI was plotted on the y-axis as a function of effect level (Fa) on the x-axis to assess the synergism, additive effect, and antagonism. The 11-point CI scale used for analysis is as follows: <0.1 very strong synergism, 0.1–0.3 strong synergism, 0.3–0.7 synergism, 0.7–0.85 moderate synergism, 0.85–0.90 slight synergism, 0.90–1.10 nearly additive, 1.10–1.20 slight antagonism, 1.20–1.45 moderate antagonism, 1.45–3.3 antagonism, 3.3–10 strong antagonism, and >10 very strong antagonism.

To evaluate molecular action mechanisms and anticancer effects of CHR and TMZ treatments in 2D-culture, U-87MG cells were treated with 25 µM doses of CHR and 250 µM doses of TMZ individually, as well as in combination, for a duration of 48-h, based on the drug synergy results. The final concentration of DMSO in the culture medium did not exceed 0.275%.

To evaluate the functional contribution of autophagy to the cellular response elicited by CHR and TMZ, pharmacological inhibition of autophagy was performed using Wortmannin (Wu et al., 2010). Cells were first incubated with 1 µM Wortmannin (#PHZ1301, Invitrogen/USA) for 6-h. Following this pre-treatment, the medium containing Wortmannin was removed and replaced with fresh medium containing CHR, TMZ, or their combination. After 48-h of treatment, cell viability was assessed by MTT assay, and Western blot analysis was performed to evaluate LC3A-II and cleaved caspase-3 protein levels as markers of autophagy and apoptosis, respectively.

### 2.3 Colony formation assay

Glioma cells’ colony-forming abilities were assessed using a 2D-colony formation assay (CFA). Post-treatment, U-87MG cells were collected, suspended in medium, plated at 1,000 cells/well in 6-well plates, and incubated at 37 °C for 7-days. Cells were then fixed with a methanol:acetic acid mixture (3:1; 5-min/37 °C), stained with 0.5% crystal-violet (15-min, Merck/Darmstadt-Germany), washed with distilled water, air-dried, and photographed. Colonies were imaged with ChemiDoc-XRS/ImageLab-6.0.1 software (Bio-Rad/Hercules-USA) and counted using ImageJ software.

### 2.4 Cell migration and invasion assays

*In vitro* scratch assay was performed to evaluate the migration of glioma cells. One-day after cell seeding (6 × 10^4^ cells/well of the 24-well culture plate), the scratch was created with the help of a 1000 µL tip, cells were washed with D-PBS and treated with CHR, TMZ, and CHR plus TMZ. Images of cells were taken at 0-h and 48-h. Additionally, Boyden chamber assay was employed to assess the quantity of invited and migrated cells. Transwell inserts with 8 μm-pore (Sarstedt/Nümbrecht-Germany), precoated with or without matrigel (50 μL, #354230, Corning/Wiesbaden-Germany), were utilized to detect invasion and migration, respectively. Treated cells (3 × 10^4^ per well in FBS-free media) were seeded in top-chamber, with 10% FBS media added to the lower chamber. After 24-h at 37 °C, invading or migrating cells were fixed with 4% PFA, stained with 0.5% crystal-violet (15-min, Merck/Darmstadt-Germany) and then photographed. Images were taken with an Olympus/CKX31 inverted light microscope and analyzed using ImageJ software.

### 2.5 Mitochondrial membrane potential assay: JC-1 staining

Mitochondrial membrane potential (MtMP) changes were assessed by JC-1 staining, where its monomers emit green-fluorescence and aggregates emit orange-red-fluorescence. After seeding (2 × 10^4^ cells/well) into 8-well chambered slides and treating with CHR and TMZ, cells were incubated with JC-1 dye loading solution (1 μg/mL, #E-CK-A301, Elabscience/Beijing-China) for 20-min/37 °C, and then washed with D-PBS. Slides were examined using a confocal-fluorescence microscope and analyzed using ImageJ software.

### 2.6 Apoptosis assay: hoechst/propidium iodide double staining

The percentage of viable, apoptotic, and dead cells were assessed by double staining with DNA-binding blue-fluorescent dye Hoechst-33342 (HO, #H-1399, Invitrogen/Carlsbad-USA) and red-fluorescent dye propidium iodide (PI, #P1304MP, Invitrogen/Carlsbad-USA). After seeding (2 × 10^4^ cells/well) into 8-well chambered slides (NuncLab-TekII, Thermo-Invitrogen/Carlsbad-USA) and treating with CHR and TMZ, cells were stained with HO/PI (5 μg/mL, 30-min/37 °C) and visualized using a confocal-fluorescence microscope (Leica-SPE2/Germany). Apoptotic and dead cells were manually counted, while ImageJ-software was used to determine total cell numbers.

### 2.7 Autophagy assay: acridine orange staining

Acidic vesicular organelles (AVO) were assessed by green-fluorescent dye acridine orange (AO) staining. After seeding (2 × 10^4^ cells/well) into 8-well chambered slides and treating with CHR and TMZ, cells were incubated with AO dye (1 μg/mL, #A1301, Invitrogen/Carlsbad-USA) for 15-min/37 °C, and then washed with D-PBS. Slides were examined to assess autophagic vacuole formation using a confocal-fluorescence microscope and analyzed using ImageJ software.

### 2.8 Immunofluorescence labeling

Intracellular localizations of NF-κB/p65 and P-glycoprotein were assessed by immunofluorescence (IF) labeling. After seeding (2 × 10^4^ cells/well) into 8-well chambered slides and treating with CHR and TMZ, cells were fixed with 4% PFA (20-min/37 °C), washed with D-PBS, and incubated with blocking-permeabilization buffer (1% BSA, 0.5% TritonX-100 in D-PBS, 1-h/RT), followed by incubation with primary (overnight/4 °C) and DyLight secondary (1-h/RT) antibodies (Table 1). Hoechst-33342 was used to stain the nuclei. The stained cells were observed using a confocal-fluorescence microscope.

**TABLE 1:** Antibodies used for Western blotting (WB) and immunofluorescence (IF) staining.

Antibody	Host	Dilution (WB)	Dilution (IF)	Catalog number	Company^a^
Anti-Bax	Rabbit	1:1,000	—	50599-2-Ig	Proteintech
Anti-Bcl-2	Rabbit	1:1,000	—	12789-1-AP	Proteintech
Anti-Cas-3/p17/p19	Rabbit	1:1,000	—	19677-1-AP	Proteintech
Anti-PARP1	Rabbit	1:1,000	—	13371-1-AP	Proteintech
Anti-LC3AI/II	Mouse	1:1,000	—	PA5-22990	Thermo/Invitrogen
Anti-Hsp60	Rabbit	1:1,000	—	15282-1-AP	Proteintech
Anti-Hsp70	Mouse	1:1,000	—	MA3-007	Thermo/Invitrogen
Anti-Hsp90	Rabbit	1:1,000	—	MA1-10372	Thermo/Invitrogen
Anti-Grp78	Rabbit	1:1,000	—	NBP2-16749	Novus
Anti-IRE1α	Rabbit	1:1,000	—	NB100-2324	Novus
Anti-ATF6	Mouse	1:1,000	—	NBP1-40256	Novus
Anti-NF-κB-p65	Rabbit	1:1,000	1:400	10745-1-AP	Proteintech
Anti-P-glycoprotein	Rabbit	1:1,000	1:400	22336-1-AP	Proteintech
Anti-MRP1	Mouse	1:1,000	—	67228-1-Ig	Proteintech
Anti-β-catenin	Rabbit	1:1,000	—	PA5-19469	Thermo/Invitrogen
Anti-E-cadherin	Rabbit	1:1,000	—	PA5-32178	Thermo/Invitrogen
Anti-N-cadherin	Mouse	1:1,000	—	MA1-91128	Thermo/Invitrogen
Anti-TGF-β	Rabbit	1:1,000	—	MA5-15065	Thermo/Invitrogen
Anti-Twist	Mouse	1:500	—	MA5-17195	Thermo/Invitrogen
Anti-CD44	Rabbit	1:1,000	—	15675-1-AP	Proteintech
Anti-CD133	Rabbit	1:1,000	—	18470-1-AP	Proteintech
Anti-Nanog	Rabbit	1:500	—	14295-1-AP	Proteintech
Anti-β-actin	Mouse	1:2,500	—	MA5-15739	Thermo/Invitrogen
Anti-Mouse IgG	Goat	1:5,000	—	31430	Thermo/Invitrogen
Anti-Rabbit IgG	Goat	1:5,000	—	31460	Thermo/Invitrogen
Anti-Rabbit IgG/DL633	Goat	-	1:400	35562	Thermo/Invitrogen

^a^ Antibodies used for immunoblotting and immunofluorescence staining were from Novus (St.Louis-USA), Proteintech (Chicago-USA), and Thermo/Invitrogen (Carlsbad-USA).

### 2.9 Protein extraction and western blot analysis

The target protein levels were analyzed by Western blotting using the standard procedure previously described (Şengelen and Önay-Uçar, 2024). After seeding (3 × 10^5^ cells/well) into 8-well chambered slides and treating with CHR and TMZ, cells were harvested, and lysed with ice-cold RIPA buffer (#89900, Thermo/Kwartsweg-Holland) supplemented with EDTA-free-PIC (1tablet/50 mL, Roche/Darmstadt-Germany) and PMSF (1 mM, AppliChem/Darmstadt-Germany). Protein concentrations were determined using BCA assay (iNtRON Biotechnology/Seongnam-Korea). Equal amounts of protein (30 μg/well) were separated via SDS-PAGE and transferred onto a PVDF membrane (Bio-Rad/Hercules-USA). Blots were probed with indicated primary (overnight/4 °C) and HRP-linked-secondary (2-h/RT) antibodies (Table 1). β-actin was used as a loading control (see Supplementary Figures S1–S5 for all reference protein images). ECL substrate (SuperSignal West-Pico, Thermo/Kwartsweg-Holland) and ChemiDoc-XRS/ImageLab-6.0.1 software (Bio-Rad, Hercules-USA) were used for visualization.

### 2.10 Growth and viability experiments in 3D glioma spheroid model

U-87MG glioma 3D-spheroids were generated using the hanging drop/agarose method, as described previously by Şengelen and Önay-Uçar (2024). Cell suspensions (2 × 10^4^ cells per 10 μL drop) were pipetted onto inverted plate lids. The lid was carefully positioned over the agarose/media-coated plate (1% final concentration), and the droplets were maintained in a hanging position under humidified conditions for 4-days to allow spheroid formation via gravity-driven cell aggregation for 4 days. Spheroids were gently transferred into wells by centrifugation (500 rpm/10-s, Eppendorf-5810R) and incubated for an additional 3-days.

Acid phosphatase (APH) assay (Friedrich et al., 2007) was performed to determine the spheroid viability and IC_50_ values of CHR (concentration range of 0–200 μM) and TMZ (concentration range of 0–2000 μM) treatments. Treated spheroids with CHR and/or TMZ were washed with D-PBS and incubated in the APH test solution (0.1 M sodium acetate, 0.1% TritonX-100, 2 mg/mL p-nitrophenyl phosphate, pH4.8) for 90-min at 37 °C. To stop the enzymatic reaction, 1N NaOH (10 µL per spheroid) was added, and absorbance at 405 nm was measured.

To evaluate the effects of CHR and TMZ treatments on spheroid growth, 3D-cultured cells were treated with 25µM and 50 µM doses of CHR and 250µM and 500 µM doses of TMZ individually, as well as in combination. The final concentration of DMSO in the culture medium did not exceed 0.550%. The impact of a single treatment was assessed after 48-h. Repeated treatment was administered once every 2 days, for a total of three treatments. Images of spheroids were taken using a digital-camera attached (ToupTek Photonics XCAM-1080PHD/Zhejiang-China) invert microscope (Olympus CKX31/Tokyo-Japan) at days 0, 2, 4 and 6. The areas of the spheroids were measured using the NIH Image J-software.

### 2.11 Bioinformatic analysis

The canonical SMILES structures of CHR and TMZ were obtained from PubChem (https://pubchem.ncbi.nlm.nih.gov/), and their potential target proteins (probability score >0) were predicted using SwissTargetPrediction (http://www.swisstargetprediction.ch/). Glioblastoma-related genes were identified through GeneCards (https://www.genecards.org). Common targets between CHR, TMZ, and glioblastoma were found via Venn diagram analysis (http://bioinformatics.psb.ugent.be/webtools/Venn/). Protein–protein interaction (PPI) networks of overlapping targets were analyzed using the STRING database v12.0 ((http://string-db.org). GO and KEGG pathway enrichment analyses were performed using ShinyGO v8.0 (http://bioinformatics.sdstate.edu/go/).

### 2.12 Statistical analysis

Data were presented as mean ± SD from at least three independent experiments. Graphs and statistical evaluations were done using GraphPad Prism program (v10.4). Nonlinear regression analysis of the sigmoidal dose-response curve was conducted to determine the IC_50_ values. Statistical analysis was performed using one-/two-way analysis of variance (ANOVA) followed by Tukey post-hoc-test. A *P* value less than 0.05 was considered statistically significant.

## 3 Results

### 3.1 Chrysin and temozolomide combined treatments synergistically reduce glioma proliferation

**FIGURE 1:**
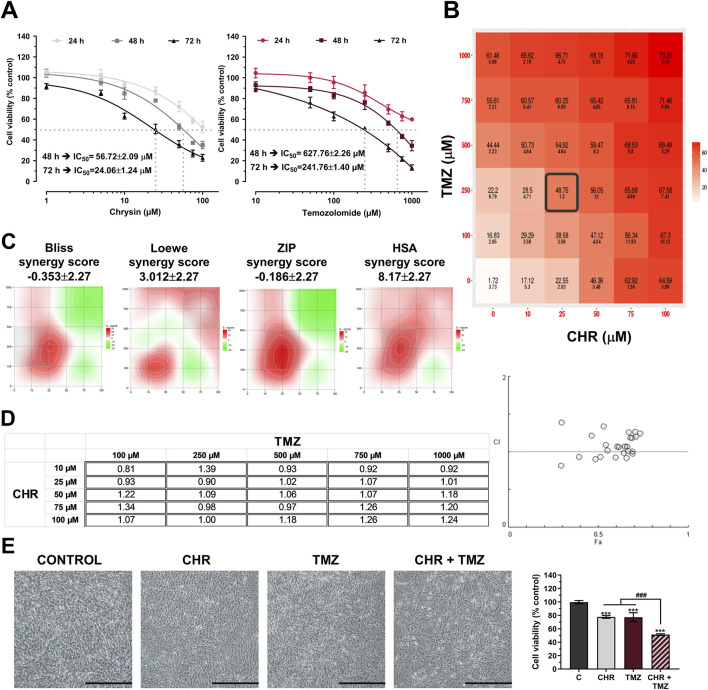
Chrysin (CHR) and temozolomide (TMZ) dose- and time-dependently reduce the viability and synergistically suppress proliferation U-87MG glioblastoma cells. **(A)** Cells were treated with increasing concentrations of CHR (0-100 µM) and TMZ (0-1000 µM) for 24-h, 48-h, and 72-h. MTT assay was performed to detect cell viability and IC_50_ (the half-maximal inhibitory concentration) values (n = 6). To calculate the IC_50_ values of treatments, a nonlinear regression analysis of the sigmoidal dose–response curve was performed. **(B)** Cells were treated with five drug doses for 48-h (n = 4) and dose-response matrices were generated to quantify synergistic interactions. **(C)** The percentage inhibition of proliferation, calculated according to the MTT results (n = 4), was used to determine the synergy score (SS) value. Heat-map responses, Bliss, Loewe, ZIP, and HSA Synergy scores were calculated using SynergyFinder software. **(D)** The CI value was determined based on the MTT results (n = 4) using Compusyn software. CI was plotted on the y-axis as a function of effect level (Fa) on the x-axis to assess the synergism, additive effect, and antagonism. **(E)** The effects of single (25 µM CHR and 250 µM TMZ) or combined (CHR + TMZ) treatments on cell morphology and viability (n = 4). Data are presented as mean ± SD. ***P < 0.001 versus control; ^###^ *P* < 0.001 versus CHR + TMZ. Statistical evaluation was performed with one-way ANOVA (Tukey post-hoc-test).

The antiproliferative effects of CHR (0-100 μM) and TMZ (0-1000 μM) treatments were assessed in U-87MG human glioblastoma cells. As indicated in Figure 1A, MTT assay demonstrated that both therapeutic agents exhibited dose- and time-dependent cytotoxicity. The cytotoxic response to CHR and TMZ was similar across all exposure periods, but the IC_50_ value for the 24-h treatment could not be calculated, suggesting limited early cytotoxicity. IC_50_ values ​​of CHR and TMZ were determined to be, respectively, 56.72 μM and 627.76 μM following a 48-h exposure, and 24.06 μM and 241.76 μM after a 72-h treatment; the decrease in IC_50_ values over time reflects a significant time-dependent increase in drug potency. Besides, CHR demonstrated higher cytotoxicity compared to TMZ at lower dose levels.

Drug synergism analysis was conducted utilizing SynergyFinder and Compusyn software to evaluate the synergistic interaction between CHR and TMZ. Cell viability inhibition percentages (quantified via MTT assay) served as the basis for calculating the combination index (CI) and synergy score (SS) to determine potential drug interactions. Dose-response matrix revealed synergistic interactions between CHR and TMZ (Figure 1B). As shown in Figure 1C, synergistic regions detected through Bliss, Loewe, ZIP, and HSA analysis were mainly found at 10-25 μM CHR and 100-250 μM TMZ concentrations. Three out of four models yielded the highest SS values for 25 µM CHR and 250 µM TMZ concentrations (22.64 for Bliss, 18.70 for ZIP, and 30.68 for HSA), while this combination ranked second in the Loewe model with 3.15 SS (the highest score belonged to 10 µM CHR +100 µM TMZ, with an SS value of 12.28). Besides, Compusyn-generated Fa-CI plot identified two synergistic doses with CI values 0.81 (for 10 µM CHR +100 µM TMZ) and 0.90 (for 25 µM CHR +250 µM TMZ) (Figure 1D). The synergy analysis results identified the combination of 25 μM CHR and 250 μM TMZ as the optimal dose for subsequent molecular studies. Furthermore, phenotype analysis via microscopy, as shown in Figure 1E, indicated that glioblastoma cell growth was suppressed by CHR and TMZ at these doses, with the combined treatment showing a more pronounced effect. Control cells (exposed to 0.275% DMSO) demonstrated typical morphology, characterized by being well spread and flattened. In contrast, treated cells exhibited noticeable alterations in shape and adhesion. The cell viability graph revealed that single-agent applications resulted in approximately a 20% reduction in viability (*P* < 0.001), whereas the CHR + TMZ combination led to about a 40% reduction (*P* < 0.001).

### 3.2 Chrysin and temozolomide combined treatments synergistically induce apoptosis and autophagy in glioma cells

**FIGURE 2:**
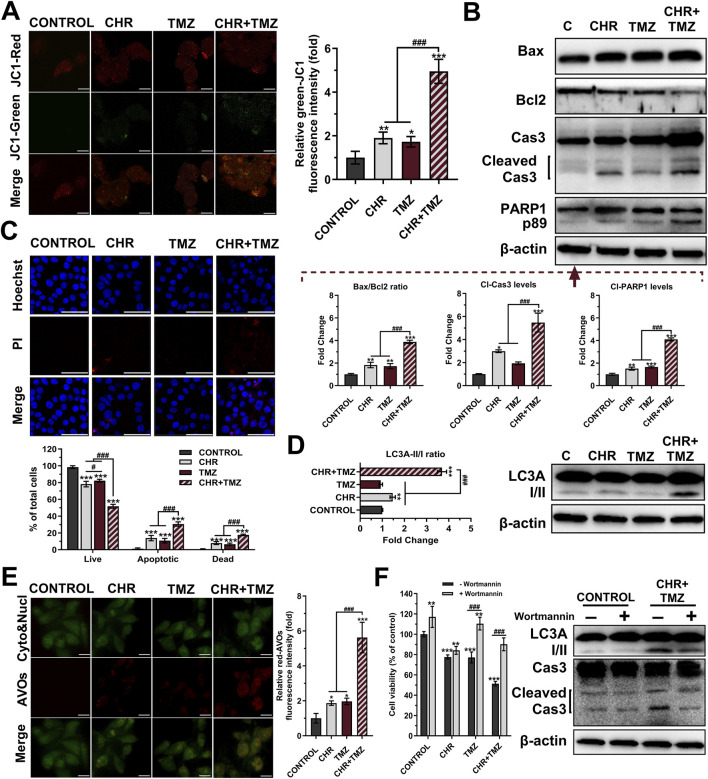
Chrysin (CHR) and temozolomide (TMZ) combination induces mitochondrial dysfunction, apoptosis, and autophagy in U-87MG glioblastoma cells. Cells were treated with CHR (25 μM), TMZ (250 μM), and CHR + TMZ for 48-h. **(A)** JC-1 staining (its monomers emit green-fluorescence and aggregates emit orange-red-fluorescence) was used to evaluate mitochondrial membrane potential (MtMP, n = 5; ×1,000, scale-bar:10 μm). **(B)** Western blot analysis (n = 3) of Bax/Bcl-2 ratio, cleaved caspase-3 (p19/17) and PARP1 (p89) levels (β-actin loading control) and quantitative data. **(C)** Double staining with Hoechst (blue-fluorescence) and PI (red-fluorescence) for determining apoptosis and quantitative analysis (n = 5; ×400, scale-bar:20 μm). **(D)** Western blot analysis (n = 3) of LC3A I/II levels (β-actin loading control) and quantitative data. **(E)** Fluorescent microscope was used to visualize the acidic vesicular organelles (AVOs, a hallmark of autophagy; red-fluorescence) as well as the cytoplasm and nucleus (green-fluorescence) after the vital staining of the cells with acridine orange (n = 5; ×1,000, scale-bar:10 μm). **(F)** To assess autophagy’s role in treatment, cells were pre-treated with an autophagy inhibitor Wortmannin (1 μM, 6-h) before exposure to CHR, TMZ, or both for 48-h. Cell viability was measured by MTT assay, and LC3A-II and cleaved caspase-3 protein levels were evaluated by Western blot. See Supplementary Figure S1 for all reference protein images and uncropped images of Western blots. Data are presented as mean ± SD. *P < 0.05, **P < 0.01 and ***P < 0.001 versus control; ^#^ *P* < 0.05 and ^###^ *P* < 0.001 versus CHR + TMZ. Statistical evaluation for HO/PI staining was performed with two-way ANOVA, while other comparisons utilized one-way ANOVA (Tukey post-hoc-test).

To assess the pro-apoptotic effects of the treatments, JC-1 staining (Figure 2A), Western blot analysis (Figure 2B) and HO/PI double staining (Figure 2C) were conducted. JC-1 staining revealed mitochondrial dysfunction through a reduced red-to-green fluorescence ratio, indicating MtMP loss. CHR and TMZ together caused more significant MtMP disruption than either treatment alone, resulting in a 4.95-fold decrease (*P* < 0.001) in mitochondrial membrane integrity in U-87MG cells. The Bax/Bcl-2 ratio exhibited a substantial increase in the CHR (1.83-fold *P* < 0.01), TMZ (1.72-fold *P* < 0.01), and CHR + TMZ (3.87-fold *P* < 0.001) groups relative to the control group. Treatment with CHR, either alone or in combination with TMZ, led to a significant elevation in cleaved-Cas3 levels (2.99-fold *P* < 0.05, and 5.46-fold *P* < 0.001, respectively). These findings indicate a disruption in MtMP status. Additionally, PARP1 p89 cleavage fragment levels were markedly increased in the CHR (1.51-fold *P* < 0.01), TMZ (1.65-fold *P* < 0.001), and CHR + TMZ (4.10-fold *P* < 0.001) groups compared to the control. Moreover, HO/PI fluorescent images showed that the CHR + TMZ combination led to more apoptotic and dead cells than CHR or TMZ alone. Hoechst-stained nuclei in controls were round and intact, while treated groups had more apoptotic bodies and nuclear condensation. Apoptosis rates were 13.84% for CHR (*P* < 0.001), 10.76% for TMZ (*P* < 0.001), and 30.60% for CHR + TMZ (*P* < 0.001) treatments. PI-positive dead cells were higher with the combination treatment, showing 7.94% for CHR (*P* < 0.001), 6.58% for TMZ (*P* < 0.001), and 17.64% for CHR + TMZ (*P* < 0.001). Findings confirmed the synergistic impact of CHR and TMZ in inducing apoptosis.

Autophagic activity was measured using Western blot analysis (Figure 2D) and AO staining (Figure 2E). At the molecular level, the LC3A-II/I ratios (indicating autophagy) were observed to be 1.47-fold (*P* < 0.01) and 3.70-fold (*P* < 0.001) higher in the CHR and CHR + TMZ groups, respectively, compared to the control group. Additionally, AVO accumulation were determined by the shift of green-fluorescence to red-fluorescence in AO assay. Treatment with CHR, TMZ, and CHR + TMZ resulted in an increase in red-AVO positive cells (1.87-fold *P* < 0.05, 1.97-fold *P* < 0.05, and 5.62-fold *P* < 0.001, respectively). To evaluate the functional role of autophagy in CHR + TMZ-induced cytotoxicity and apoptosis, U-87MG cells were pre-treated with Wortmannin (1 μM, 6-h) prior to exposure to CHR, TMZ, or their combination for 48-h. As shown in Figure 2F, MTT assay results indicated that Wortmannin pre-treatment was associated with increased cell viability in the control group (*P* < 0.01). In the CHR group, Wortmannin did not significantly alter cell viability. However, higher cell viability was noted in both the TMZ and CHR + TMZ groups following Wortmannin treatment (*P* < 0.001), suggesting that inhibition of autophagy was related to reduced cytotoxicity from these treatments. Western blot analysis aligned with these findings, showing decreased LC3A-II and cleaved caspase-3 levels in the CHR + TMZ group after Wortmannin pre-treatment. These observations suggest that the combined CHR + TMZ treatment induces autophagy more effectively than single-agent treatments.

### 3.3 Chrysin and temozolomide combined treatments synergistically change cellular stress response and drug resistance profile in glioma cells

**FIGURE 3:**
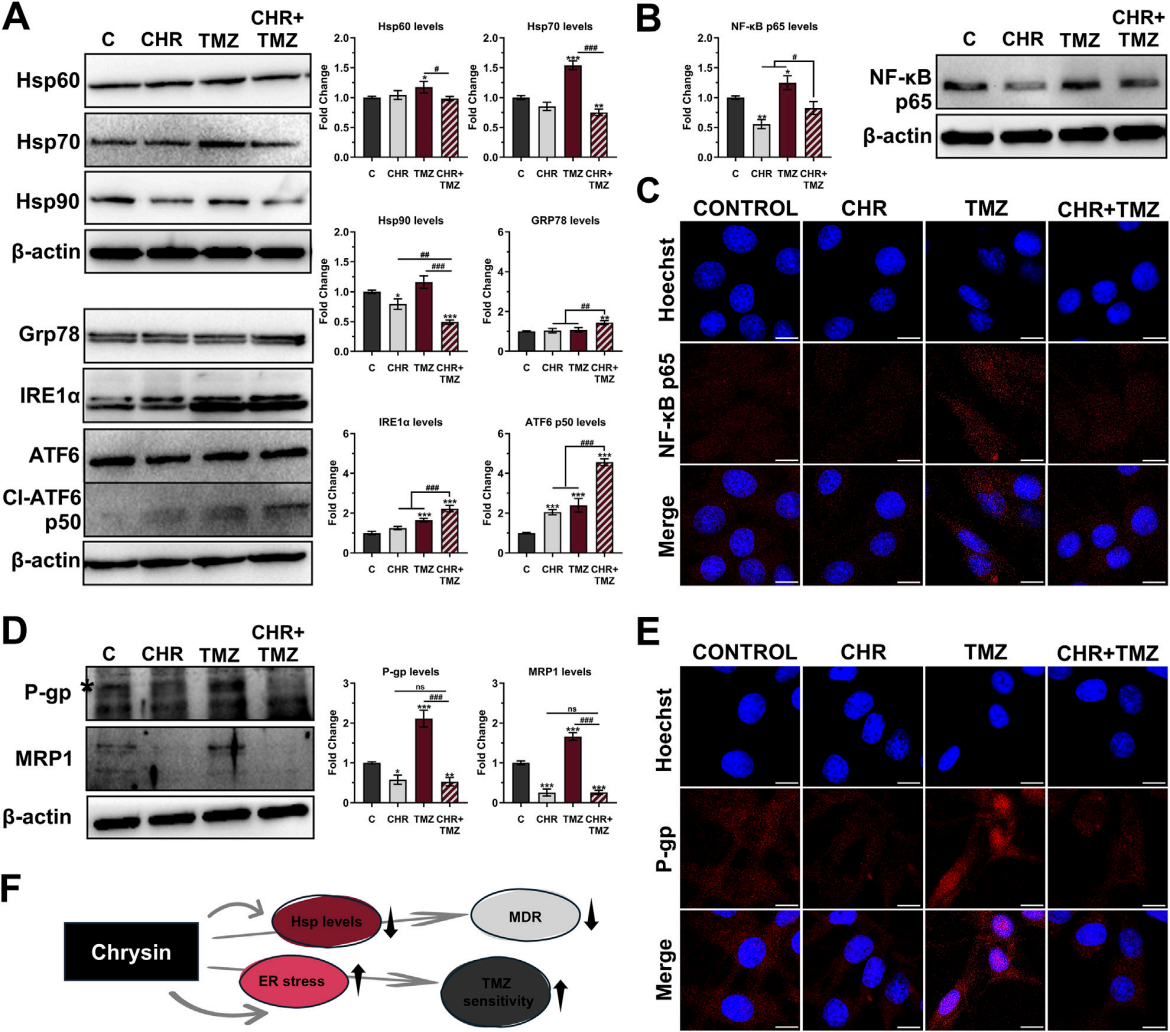
Chrysin (CHR) enhances temozolomide (TMZ) efficacy by modulating stress response, NF-κB signaling, and multidrug resistance (MDR) in U-87MG glioblastoma cells. Cells were treated with CHR (25 μM), TMZ (250 μM), and CHR + TMZ for 48-h. **(A)** Western blot analysis (n = 3) of Hsp60, Hsp70, Hsp90, GRP78, IRE1α, and cleaved ATF6 (p50) levels (β-actin loading control) and quantitative data. **(B)** Western blot analysis (n = 3) of NF-κB-p65 (β-actin loading control) and quantitative data. **(C)** Immunofluorescence labeling for NF-κB-p65 protein (*n* = 3; ×1,000, scale-bar:10 μm). **(D)** Western blot analysis (*n* = 3) of the P-gp (*a mature 170-kDa product involved in drug efflux) and MRP1 levels (β-actin loading control) and quantitative data. **(E)** Immunofluorescence labeling for P-gp (*n* = 3; ×1,000, scale-bar:10 μm). **(F)** Shape showing status Hsp levels, ER stress, MDR, and TMZ sensitivity upon CHR treatment. See Supplementary Figures S2-S4 for all reference protein images and uncropped images of Western blots. Data are presented as mean ± SD. *P < 0.05, **P < 0.01 and ***P < 0.001 versus control; ^#^ *P* < 0.05, ^##^ *P* < 0.01 and ^###^ *P* < 0.001 versus CHR + TMZ. Statistical evaluation was performed with one-way ANOVA (Tukey post-hoc-test).

As shown in Figure 3A, Western blot analysis revealed that treatment with TMZ led to a slight increase in Hsp60 levels (1.18-fold *P* < 0.05) and a robust upregulation of Hsp70 (1.54-fold *P* < 0.01), but with no difference in Hsp90 levels (*P* > 0.05). In contrast, CHR treatment prevented the TMZ-induced increase in Hsp levels in glioma cells. Co-treatment with CHR significantly reduces the levels of Hsp60 (1.19-fold *P* < 0.05), Hsp70 (2.05-fold *P* < 0.001), and Hsp90 (2.37-fold *P* < 0.001) compared to the TMZ group. Besides, the levels of Grp78, IRE1α, and ATF6-p50 were evaluated to assess ER stress in glioma cells. CHR alone did not alter Grp78 and IRE1α levels (*P* > 0.05) but increased ATF6-p50 levels (*P* < 0.001). TMZ alone treatment increased IRE1α and ATF6-p50 levels (*P* < 0.001), with no change in Grp78 levels (*P* > 0.05). However, the combination of CHR and TMZ significantly elevated levels of Grp78 (1.44-fold *P* < 0.01), IRE1α (2.22-fold *P* < 0.001), and Cl-ATF6 (4.56-fold *P* < 0.001). These findings indicate that ER stress was notably higher with CHR + TMZ co-treatment compared to either treatment alone.

Expression levels and nuclear localization of the NF-κB-p65 transcription factor were assessed using immunoblotting (Figure 3B) and immunofluorescence labeling (Figure 3C) analysis. CHR treatment led to a significant decrease in p65 expression (1.80-fold *P* < 0.01), whereas TMZ treatment increased its levels (1.25-fold *P* < 0.05). Notably, the elevated levels induced by TMZ were significantly suppressed by co-treatment with CHR, resulting in a 1.51-fold decrease (*P* < 0.05). Consistently, immunofluorescence labeling revealed increased nuclear translocation of NF-κB-p65 in glioma cells treated with TMZ alone. In contrast, a substantial reduction in nuclear localization was observed following combination treatment with CHR.

To assess the impact of treatments on drug resistance mechanisms in U-87MG glioma cells, the expression levels of the key resistance markers P-glycoprotein (P-gp) and multidrug resistance-associated protein 1 (MRP1) were evaluated using immunoblotting (Figure 3D) and immunofluorescence analysis (Figure 3E). Notably, exposure to TMZ alone significantly elevated the levels of P-gp (2.11-fold *P* < 0.001) and MRP1 (1.66-fold *P* < 0.001) compared to control cells. Conversely, CHR treatment inhibited the TMZ-induced increase in MDR protein levels. Specifically, P-gp expression (a mature 170-kDa product involved in drug efflux) was markedly reduced following CHR (1.74-fold *P* < 0.05) and CHR + TMZ (1.91-fold *P* < 0.01) treatments. Similarly, MRP1 levels were significantly downregulated in the CHR (3.90-fold *P* < 0.001) and CHR + TMZ (3.88-fold *P* < 0.001) groups. Importantly, combined treatment resulted in a substantial reduction in P-gp (4.04-fold *P* < 0.001) and MRP1 (6.44-fold *P* < 0.001) expressions compared to treatment with TMZ alone. Additionally, immunofluorescence analysis showed that P-gp was highly localized at the nuclear membrane in the TMZ-only treatment group. However, co-treatment with CHR significantly diminished both the overall expression of P-gp and its localization to the nuclear membrane.

As briefly stated in Figure 3F, CHR not only suppressed Hsp and MDR marker levels but also induced ER stress, thereby enhancing the cells’ sensitivity to TMZ.

### 3.4 Chrysin and temozolomide combined treatments synergistically decrease colony formation, migratory, and differentiation characteristics of glioma cells

**FIGURE 4:**
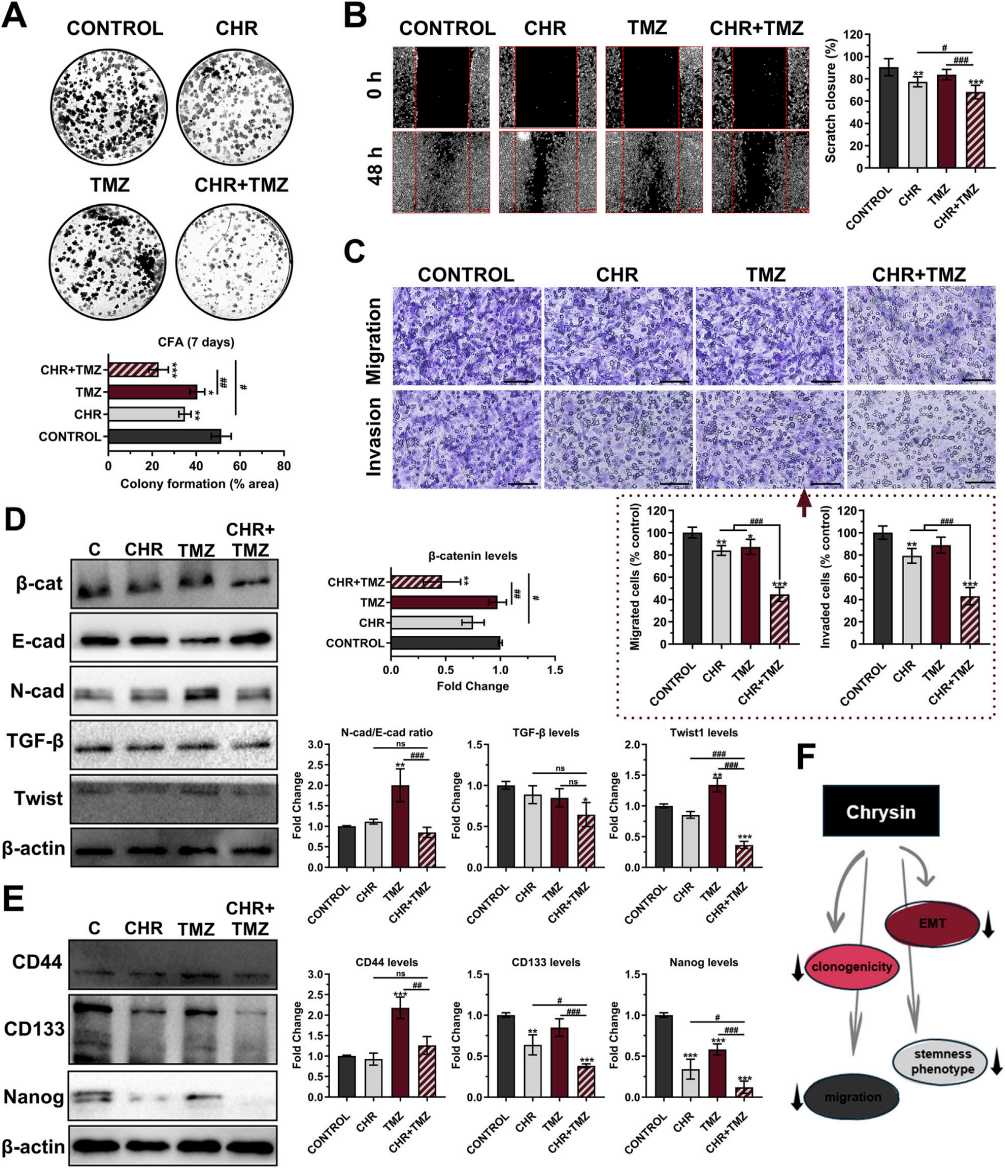
Chrysin (CHR) in combination with temozolomide (TMZ) synergistically suppresses clonogenicity, migration, epithelial-to-mesenchymal transition (EMT), and stemness features in U-87MG glioblastoma cells. Cells were treated with CHR (25 μM), TMZ (250 μM), and CHR + TMZ for 48-h. **(A)** The colony-forming ability of the cells was evaluated with CFA in 2D-culture (crystal violet staining on day 7; n = 3) **(B)** The migratory ability of the cells was evaluated by scratch-wound assay (n = 6; ×40, scale-bar:400 µm). **(C)** Transwell migration and invasion assay (n = 5; ×200, scale-bar:200 µm). **(D)** Western blot analysis (n = 3) of EMT markers N-cadherin/E-cadherin, β-catenin, Twist1, TGF-β (β-actin loading control) and quantitative data. **(E)** Western blot analysis (n = 3) of cancer stem cell (CSC) markers CD44, CD133, and Nanog (β-actin loading control) and quantitative data. **(F)** Shape showing decreased clonogenicity, migration, EMT, and CSC status upon CHR treatment. See Supplementary Figure S5 for all reference protein images and uncropped images of Western blots. Data are presented as mean ± SD. *P < 0.05, **P < 0.01 and ***P < 0.001 versus control; ^#^ *P* < 0.05, ^##^ *P* < 0.01 and ^###^ *P* < 0.001 versus CHR + TMZ. Statistical evaluation was performed with one-way ANOVA (Tukey post-hoc-test).

The clonogenic potential of U-87MG glioma cells was evaluated by CFA (Figure 4A). Colony-forming ability decreased by 1.47-fold (*P* < 0.01) with CHR, 1.27-fold (*P* < 0.05) with TMZ, and 2.24-fold (*P* < 0.001) with CHR + TMZ compared to the control group; suggesting that the CHR + TMZ combination more effectively reduces colony formation in glioma cells than either treatment alone. As shown in Figure 4B, the scratch migration assay revealed that TMZ treatment did not reduce the migratory capacity of glioma cells (*P* > 0.05), while CHR (1.17-fold *P* < 0.01) and CHR + TMZ (1.33-fold *P* < 0.001) treatments significantly reduced the rate of wound closure. Additionally, the transwell chamber assay (Figure 4C) revealed a significant reduction in glioma cell migration and invasion after treatment. In the CHR group, migrated and invaded cells were 87.08% (*P* < 0.01) and 79.21% (*P* < 0.01) of control levels, respectively. In the CHR + TMZ group, these values decreased to 44.69% (*P* < 0.001) and 43.00% (*P* < 0.001), respectively.

Epithelial-mesenchymal transition (EMT) was evaluated by immunoblotting to examine the levels of EMT markers, including β-catenin, E-cadherin, N-cadherin, TGF-β, and Twist1 (Figure 4D). β-catenin expression decreased after CHR + TMZ treatments (2.14-fold *P* < 0.01) compared to the control. TMZ exposure increased the N-cadherin (a mesenchymal marker)/E-cadherin (an epithelial marker) ratio (2.00-fold *P* < 0.01) and Twist1 levels (2.74-fold *P* < 0.001) compared to the control, indicating an enhanced EMT phenotype and resistance to drug therapy. Co-treatment with CHR prevented these increases; N-cadherin/E-cadherin ratio and Twist1 levels were reduced by 2.36-fold (*P* < 0.001) and 3.72-fold (*P* < 0.001) compared to the TMZ group. Additionally, CHR + TMZ treatment also decreased TGF-β expression (1.55-fold *P* < 0.05) compared to the control. In addition to evaluating EMT, the differentiation properties of glioma cells were assessed by determining the levels of cancer stem cell (CSC) markers CD44, CD133, and Nanog (Figure 4E). The CHR treatment resulted in a significant decrease in CD133 (1.57-fold *P* < 0.01) and Nanog expression (2.93-fold *P* < 0.001) but did not affect CD44 levels (*P* > 0.05) compared to the control group. Conversely, TMZ treatment significantly increased CD44 levels (2.18-fold *P* < 0.001) while having no significant effect on CD133 levels (*P* > 0.05) and causing a reduction in Nanog expression (1.72-fold *P* < 0.001). Notably, CHR and TMZ co-treatment decreased these marker levels compared to both the control (except for CD44) and TMZ groups; this treatment resulted in a 2.62-fold decrease (*P* < 0.001) in CD133 and an 8.29-fold decrease (*P* < 0.001) in Nanog levels compared to the control group. Our findings demonstrated that the CHR + TMZ combination treatment was more effective in suppressing EMT and CSC-like properties in glioma cells than CHR or TMZ alone.

As briefly stated in Figure 4F, CHR markedly suppressed clonogenicity, migration, EMT, and stemness phenotype of glioma cells.

### 3.5 Chrysin and temozolomide combined treatments synergistically reduce viability and growth of glioma spheroids

**FIGURE 5:**
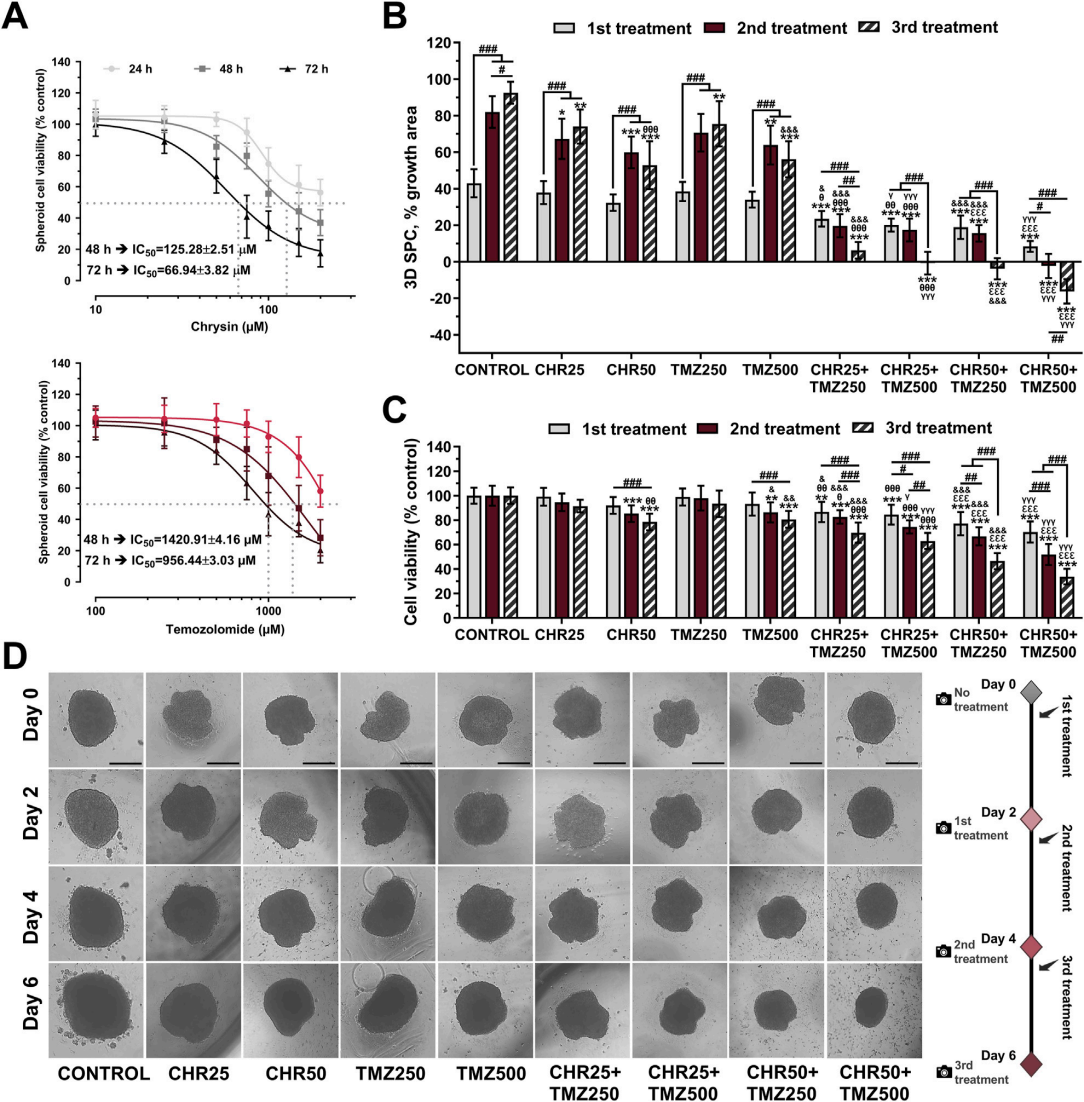
Chrysin (CHR) enhances the anti-tumor efficacy of temozolomide (TMZ) in U-87MG glioblastoma 3D-spheroid culture (SPC) model. **(A)** Spheroids were treated with increasing concentrations (10–200 μM) of CHR and (100–2000 μM) TMZ for 24-h, 48-h, and 72-h. APH assay was performed to detect cell viability and IC_50_ (the half-maximal inhibitory concentration) values (n = 10). To calculate the IC_50_ values of treatments, a nonlinear regression analysis of the sigmoidal dose–response curve was performed. **(B)** The size of spheroids was analyzed to determine spheroid growth (n = 6). **(C)** Cell viability was assessed with the APH test during the 1st, 2nd, and 3rd treatments (n = 10). **(D)** The first treatment was applied to spheroids on Day-0. The spheroids were treated with 25 μM and 50 μM of CHR, 250 μM and 500 μM of TMZ and combinations doses, photographed 48-h later, and exposed to three times (on Day-0, Day-2, and Day-4) (×40, scale-bar:400 µm). Data are presented as mean ± SD. *P < 0.05, **P < 0.01 and ***P < 0.001 versus control; ^#^ *P* < 0.05, ^##^ *P* < 0.01 and ^###^ *P* < 0.001 indicate a comparison between doses of the same group; ^θ^ *P*<0.05, ^θθ^ *P*<0.01 and ^θθθ^ *P*<0.001 versus CHR25; ^εεε^ *P*<0.001 versus CHR50; ^&^ *P* < 0.05, ^&&^ *P* < 0.01 and ^&&&^ *P* < 0.001 versus TMZ25; ^γ^ *P*<0.05 and ^γγγ^ *P*<0.001 versus TMZ50. Statistical evaluation was performed with two-way ANOVA (Tukey post-hoc-test).

CHR and TMZ treatments were observed to reduce the viability of U-87MG glioma 3D-spheroids in a time- and dose-dependent manner (Figure 5A). The IC_50_ values of CHR were 125.28 μM at 48-h and 66.94 μM at 72-h, while those of TMZ were 1,420.91 μM at 48-h and 956.44 μM at 72-h, which were approximately two-fold higher compared to the 2D-monolayer culture.

In the 3D glioma spheroid model, combinations of CHR (25 μM and 50 μM) with TMZ (250 μM and 500 μM) were evaluated for spheroid growth (Figure 5B), viability (Figure 5C), and morphology (Figure 5D). Spheroid growth in the control group increased by 42.99% after the first treatment, 81.98% after the second, and 92.57% after the third compared to day 0. CHR (50 μM) and TMZ (500 μM) halted spheroid growth by the third treatment and significantly reduced viability (CHR: 21.48% *P* < 0.001; TMZ: 19.44% *P* < 0.001). Combining the highest doses of CHR (50 μM) and TMZ (500 μM) stopped spheroid growth; it grew only 8.39% by day 2 and decreased in size on days 4 and 6. This combination yielded a 16% reduction in size and nearly 60% decrease in viability by the third treatment, showing CHR boosts TMZ efficacy in 3D spheroid cultures. Besides, control spheroids treated with the medium were compact, and migratory colonies were observed on days 2, 4, and 6. The diameter of CHR-treated spheroids was smaller compared to the control, varying based on the dose and treatment type (single or repeated). Additionally, spheroids treated with CHR showed disrupted structure with many disassociated cells, particularly in the CHR50+TMZ250 and CHR50+TMZ500 groups. These findings indicate that CHR markedly potentiated the therapeutic efficacy of TMZ in 3D spheroid cultures, as evidenced by the substantial reduction in both spheroid growth and cell viability in the combined treatment group compared to TMZ alone; thus, suggesting that CHR may enhance TMZ sensitivity and improve its antitumor activity under more physiologically relevant culture conditions.

### 3.6 Chrysin, temozolomide and glioma-related targets and their functional classifications

**FIGURE 6:**
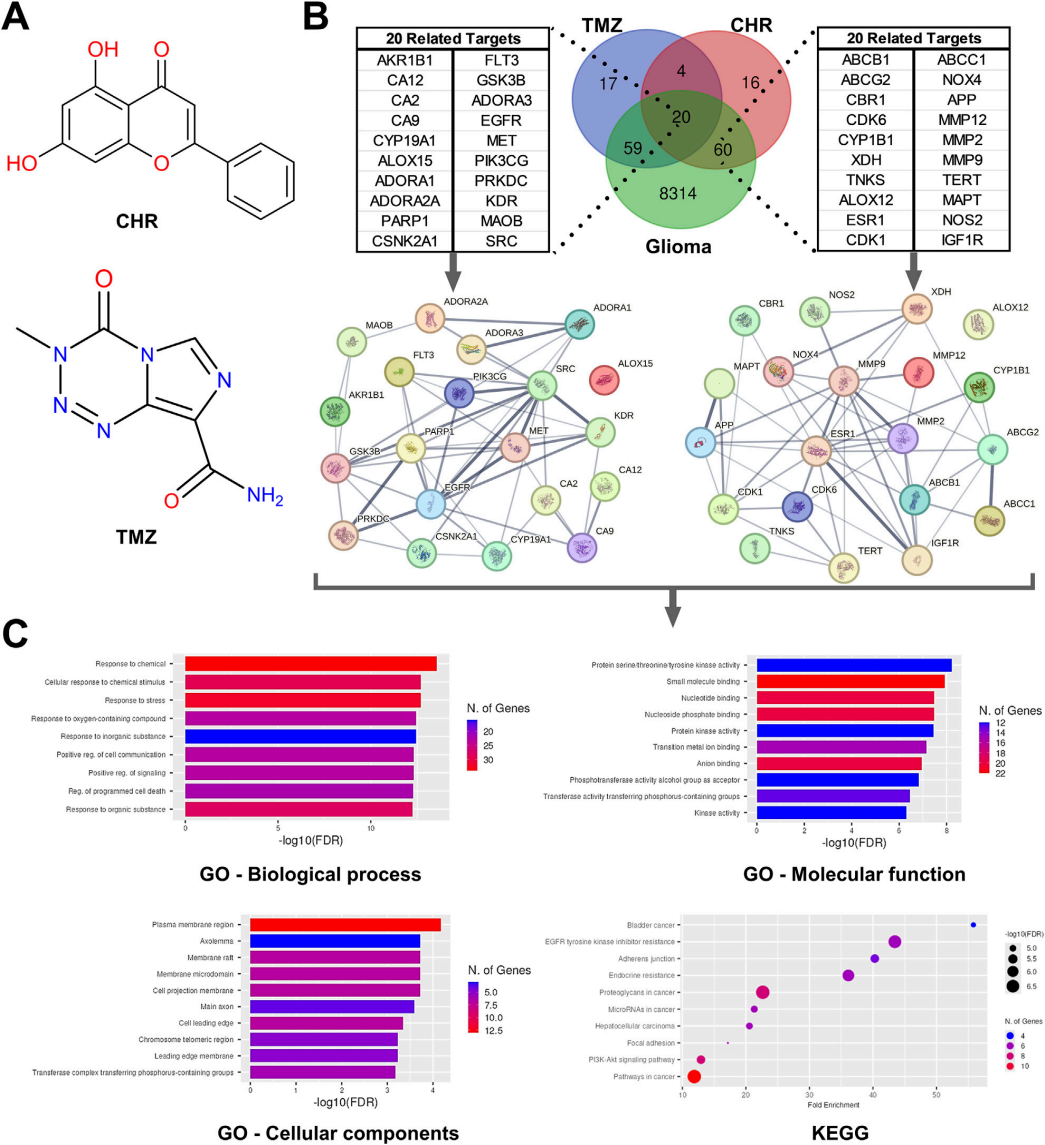
Network pharmacology analysis for predicting the possible intersection of target genes related to Chrysin (CHR), temozolomide (TMZ), and glioma. **(A)** The molecular structure of CHR and TMZ. **(B)** Venn diagram identifying the intersection of CHR, TMZ, and glioma-related target genes. The protein–protein interaction (PPI) network detected by STRING software using intersecting target genes. **(C)** The result of Gene ontology (GO; the terms are biological process, cellular components, and molecular function) and Kyoto Encyclopedia of Genes and Genomes (KEGG) pathway enrichment analyses.

The potential target proteins of CHR and TMZ were evaluated using canonical SMILES structures (Figure 6A), and 24 overlapping targets were identified, with 20 of these also being associated with glioma. Among these shared targets, several key genes involved in cancer-related pathways were identified, including EGFR, PARP1, SRC, MET, GSK3B, and CYP19A1. These genes are known to play critical roles in cell proliferation, DNA repair, and oncogenic signaling, indicating their potential as mediators of the combined anti-glioblastoma effect of CHR and TMZ. Additionally, a broader set of 60 glioma-related targets specific to CHR was identified (Figure 6B); this gene set includes multiple multidrug resistance-associated transporters (ABCB1, ABCC1, ABCG2), cyclin-dependent kinases (CDK1, CDK6), matrix metalloproteinases (MMP2, MMP9, MMP12), inflammatory mediators (NOS2, NOX4), and genes associated with glioma stemness or progression (APP, MAPT, IGF1R, TERT). According to GO and KEGG analysis (Figure 6C), these targets are involved in processes such as drug resistance, cell cycle regulation, invasion, oxidative stress, and stem cell maintenance. Functional enrichment analysis links them to responses to chemicals and inorganic substances, as well as programmed cell death regulation and cellular stress response. Plasma membrane regions, membrane rafts, and axonal structures are highlighted as key components in cellular components. Significant molecular functions include kinase activity and small molecule binding, with KEGG pathways noting the PI3K-Akt signaling pathway, EGFR inhibitor resistance, and cancer-related proteoglycan and microRNA pathways.

## 4 Discussion

Utilizing natural compounds to improve the efficacy and minimize side effects of temozolomide (TMZ) is a crucial approach (Zhai et al., 2021). Studies indicate that flavonoid chrysin (CHR) has time- and dose-dependent cytotoxicity on glioma cells (Weng et al., 2005; Liao et al., 2014; Jia et al., 2015; Santos et al., 2015; Wang et al., 2018), aligning with our cell viability results (Figure 1A/left-graph). Although TMZ is applied clinically, research continues to optimize its effectiveness (Mishchenko et al., 2025). Our findings confirm that the cytotoxic effects of TMZ are both time- and dosage-dependent, corroborated by our previous work (Şengelen and Önay-Uçar, 2024) and other studies (Lan et al., 2016; Kim et al., 2018) (Figure 1A/right-graph). SynergyFinder and Compusyn software were utilized to assess the combined impacts of CHR and TMZ; analyses showed a synergistic effect at 25 μM CHR and 250 μM TMZ (Figures 1B–D). Notably, Liao et al. (2014) showed enhanced effects of combining CHR (20 μM) and TMZ (100 μM) in GBM8901 cells (aligns with our synergistic findings), though synergy and mechanisms were not detailed. In comparison, our study investigates CHR + TMZ synergy in 2D/3D U-87MG glioblastoma models, examining its impact on apoptosis, autophagy, ER stress, EMT, and MDR, and aims to clarify its translational relevance by focusing on the mechanisms underlying the synergistic effect.

The main objective in cancer treatment is to promote apoptosis in malignant cells (Obrador et al., 2024). The study demonstrates that CHR markedly induces apoptosis, as evidenced by changes in MtMP status (Figure 2A), Bax/Bcl-2 ratio, active caspase-3 levels (Figure 2B), and HO/PI staining (Figure 2C). Furthermore, combining CHR with TMZ leads to enhanced glioma cell death through synergistic action. Prior research has documented CHR’s pro-apoptotic effects in various cancers, including bladder (Xu et al., 2018), breast (Lirdprapamongkol et al., 2013), brain (Mahdi et al., 2023), ovary (Lim et al., 2018), colon (Lin et al., 2018), gastric (Zhong et al., 2020; Lee et al., 2021), liver (Xu et al., 2017), and prostate (Ryu et al., 2017). As an apoptosis indicator, CHR plus TMZ treatment also increased cleavage of PARP1 (which plays a critical role in DNA repair) (D'Amours et al., 2001), aligning with previous findings that CHR inhibits PARP in colorectal cancer (Lin et al., 2018) and hepatocellular carcinoma (Xu et al., 2017). Supportively, CHR has been documented to enhance apoptotic responses to various chemotherapies, such as cisplatin (Raina et al., 2023), 5-FU (Lee et al., 2021), docetaxel (Lim et al., 2017), and doxorubicin (Gao et al., 2013).

Autophagy is crucial for cancer cell survival and death, with autophagy being linked to TMZ resistance in gliomas (Singh et al., 2021). However, excessive autophagy with TMZ and adjuvant treatments can enhance its therapeutic effectiveness (Yan et al., 2016). Immunoblotting (Figure 2D) and AO staining (Figure 2E) indicated an increase in autophagy following TMZ treatment compared to basal control levels; this relatively modest increase in autophagy may have diminished the TMZ’s efficacy through drug resistance. On the contrary, CHR co-treatment caused a pronounced rise in autophagy markers (LC3A-II and AVOs), suggesting excessive autophagy may drive cell death. Our findings align with studies on CHR-induced autophagy in colorectal (Lin et al., 2018) and pancreatic cancers (Zhou et al., 2021), indicating CHR could enhance TMZ-induced apoptosis through autophagy. Furthermore, wortmannin pre-treatment reversed the cytotoxic effects of CHR + TMZ, lowering LC3A-II and cleaved caspase-3 levels (Figure 2F). These results support autophagy as a pro-death mechanism facilitating apoptosis (Yan et al., 2016; Xia et al., 2020), and suggest modulating autophagy may improve responses to TMZ-based therapies in glioblastoma.

Stress proteins help cancer cells resist drugs (Somu et al., 2023). While the impact of CHR on Hsp levels in cancer cells remains unknown, TMZ has been shown to increase Hsp expression (Castro et al., 2015). In this study, CHR did not affect Hsp expression, except for lowering Hsp90 levels. CHR also reduced TMZ-induced Hsp upregulation while boosting ER stress (Figure 3A), similar to its effects in prostate (Ryu et al., 2017) and bladder cancer (Xu et al., 2018). Moreover, NF-κB is a critical transcription factor that regulates the inflammation, survival, anti-apoptotic, and MDR responses of cancer cells (DiDonato et al., 2012; Sui et al., 2014). CHR co-treatment reduced TMZ-induced NF-κB p65 levels and nuclear localization, as evidenced by immunoblotting (Figure 3B) and immunofluorescence labeling (Figure 3C), suggesting enhanced TMZ efficacy via NF-κB inhibition. Additionally, TMZ significantly upregulated P-gp (ABCB1) and MRP1 (ABCC1) transporter proteins, whereas CHR alone or with TMZ suppressed these increases (Figure 3D) and reduced P-gp nuclear localization (Figure 3E). Given that ATP-dependent membrane transporters such as P-gp and MRP1 mediate drug efflux and limit chemotherapeutic efficacy (Gottesman et al., 2002; Robey et al., 2018), these findings indicate CHR’s potential to counteract TMZ-induced MDR, a notable factor in treatment resistance in gliomas (Rich and Bigner, 2004). Decreased expression of these transporters by CHR may boost TMZ’s cytotoxicity by increasing its intracellular concentration. Flavonoids have been reported to inhibit P-gp in various cancers (Kumar et al., 2023), and P-gp blockade has been shown to sensitize glioma cells to temozolomide (Yu et al., 2024). Additionally, blocking the NF-κB pathway enhances TMZ efficacy in glioblastoma (Yu et al., 2018). Supportively, CHR has also been reported to suppress MDR transporters (Brechbuhl et al., 2012) and to inhibit NF-κB signaling in various cancer models (Wu et al., 2018; Yeo et al., 2020). Collectively, these previous reports and our findings reveal that the suppressive effect of CHR on MDR-associated transporters is a crucial mechanism for increasing TMZ sensitivity alongside changes in cellular stress responses (Figure 3F).

The combination of CHR and TMZ significantly inhibited U-87MG glioblastoma cell colony formation capacity (Figure 4A), migration and invasion (Figure 4B,C), EMT (Figure 4D), and CSC properties (Figure 4E), thereby reducing long-term proliferative capacity and impairing key drivers of tumor aggressiveness (Wang et al., 2018). Supportively, research reveals that CHR reduces colony formation and invasiveness in various cancers, including cervix/ovary (Lim et al., 2018; Raina et al., 2021), brain (Wang et al., 2018), breast (Yang et al., 2014), gastric (Zhong et al., 2020), and lung (Tang et al., 2024). These effects were linked to downregulation of EMT markers such as β-catenin, N-cadherin, TGF-β, and Twist1, while E-cadherin levels remained stable, indicating preserved epithelial characteristics and inhibition of mesenchymal transition (Lee et al., 2014); this is supported by CHR’s reducing impact on the EMT status of breast cancer cells (Yang et al., 2014). Furthermore, the CHR + TMZ combination also significantly reduced the expression of stem cell markers CD133 and Nanog (Deldar Abad Paskeh et al., 2021), and countered TMZ-induced increases in CD44, suggesting CHR mitigates glioma progression by reducing stem-cell characteristics (Raina et al., 2024). Overall, CHR enhances tumor cell sensitivity to TMZ and inhibits the migrative-invasive, stem cell-like, and EMT-related properties of glioblastoma cells, offering a multifaceted approach to combat tumor progression (Figure 4F).

Recent interest in 3D-spheroid models stems from their more accurate simulation of the tumor microenvironment, capturing features like hypoxia, nutrient gradients, and cell-cell interactions (Kapałczyńska et al., 2018). In this study, U-87MG glioblastoma spheroids showed roughly double IC_50_ values for CHR and TMZ compared to 2D-cultures, suggesting greater drug resistance in 3D conditions (Figure 5A), consistent with both our previous findings (Şengelen and Önay-Uçar, 2024) and existing literature (Nunes et al., 2019). Despite TMZ being the standard chemotherapy for glioblastoma, resistance remains a significant challenge for effective treatment (Stupp et al., 2005). As shown in Figures 5B–D, high doses of TMZ reduced spheroid growth and viability, and its combination with CHR further enhanced these effects. Repeated treatment significantly inhibited spheroid growth and even reduced spheroid size. Notably, 50 μM CHR plus 500 μM TMZ produced a pronounced synergistic reduction in growth and viability, consistent with reports that flavonoid–chemotherapy combinations suppress proliferation in 3D glioma models (Zhai et al., 2021). Supportively, CHR’s tumor size reduction has been observed in brain (Wang et al., 2018), gastric (Zhong et al., 2020), liver (Xu et al., 2017), lung (Tang et al., 2024), and pancreatic (Zhou et al., 2021) cancers. Therefore, combining CHR with TMZ may improve chemotherapy efficacy and decrease drug resistance in glioblastoma, but further *in vivo* and clinical studies are needed to explore this potential.

Our findings on cell motility, EMT/stem-like state, MDR, and apoptosis were supported by bioinformatics and network pharmacology analyses (Figure 6), suggesting CHR can mitigate drug resistance and EMT-CSC profiles observed in glioma spheroids from our previous study (Şengelen and Önay-Uçar, 2024). Twenty common targets, including EGFR, PARP1, SRC, MET, GSK3B, and CYP19A1, were identified, all crucial regulators of proliferation, DNA repair, and oncogenic signaling (Gan et al., 2009; Murai et al., 2012; Li et al., 2023). Sixty glioma-associated targets were associated with CHR, such as MDR transporters (ABCB1, ABCC1, ABCG2), cyclin-dependent kinases (CDK1, CDK6), matrix metalloproteinases (MMP2, MMP9, MMP12), and genes related to inflammation or oxidative stress (NOS2, NOX4) (Levicar et al., 2003; Agarwal et al., 2010; Gialeli et al., 2011). Functional enrichment analysis highlighted roles in chemical response, cell death, cellular stress, and membrane- and axon-associated components, and kinase and small molecule binding. KEGG analysis revealed PI3K-Akt signaling, EGFR inhibitor resistance, and other cancer-related pathways (Manning et al., 2002; Caretta and Mucignat-Caretta, 2011; Li et al., 2022).

Consequently, this study is the first to report on the potential anticancer mechanism of CHR plus TMZ treatment in 2D- and 3D-cultured gliomas, but it has some limitations. Due to genetic diversity among GB patients, using various cell lines may provide more generalizable results (Xie et al., 2014). Although spheroid culture mimics *in vivo* environment, it does not encompass the complex tumor microenvironment, immune response, vascularization, and pharmacokinetics; animal studies are needed to elucidate bioavailability, systemic toxicity, and therapeutic index of the CHR and TMZ combination (Hottinger et al., 2016). Additionally, targeted drug delivery systems like nanoparticles (Mahdi et al., 2023; Oliyapour et al., 2023) may improve efficacy by enhancing CHR’s bioavailability and solubility. Another limitation is the lack of functional validation, such as gene silencing or pharmacological inhibition, to confirm the roles of P-gp and NF-κB pathways. Pathway-specific inhibition studies are also needed to clarify the effects of treatment-induced ER stress on cytotoxicity and apoptosis. Future research will address these gaps for stronger mechanistic insights. Despite these limitations, the findings support further research into CHR, which enhances TMZ’s effectiveness and may serve as a useful adjuvant therapy for gliomas. In-depth studies could confirm CHR plus TMZ as a promising approach to treating GB cancers.

## Data Availability

The raw data supporting the conclusions of this article will be made available by the authors, without undue reservation.

## Abbreviations

AVOs, acidic vesicular organelles; CFA, colony formation assay; CHR, Chrysin; CI, combination index; CSC, cancer stem cell; EMT, epithelial-mesenchymal transition; ER, endoplasmic reticulum; GB, Glioblastoma; HO/PI, Hoechst/Propidium iodide; Hsp, Heat shock protein; IC50, Half maximal inhibitory concentration; MDR, multidrug resistance; MRP1, multidrug resistance-associated protein 1; MtMP, Mitochondrial membrane potential; NF-κB, nuclear factor-kappaB; P-gp, P-glycoprotein; SS, synergy score; TMZ, Temozolomide.
