# Supplementary material for: Chrysin sensitizes glioblastoma cells and spheroids to temozolomide treatment by reducing EMT and stemness phenotypes, as well as targeting multidrug resistance proteins
Source: Front Pharmacol. 2025 Sep 2;16:1643186. doi: 10.3389/fphar.2025.1643186 (PMC12436294; doi:10.3389/fphar.2025.1643186)

Supplementary Material

**Figure S1:** Uncropped images of blots and all reference loading controls in Fig.2B and Fig.2D.


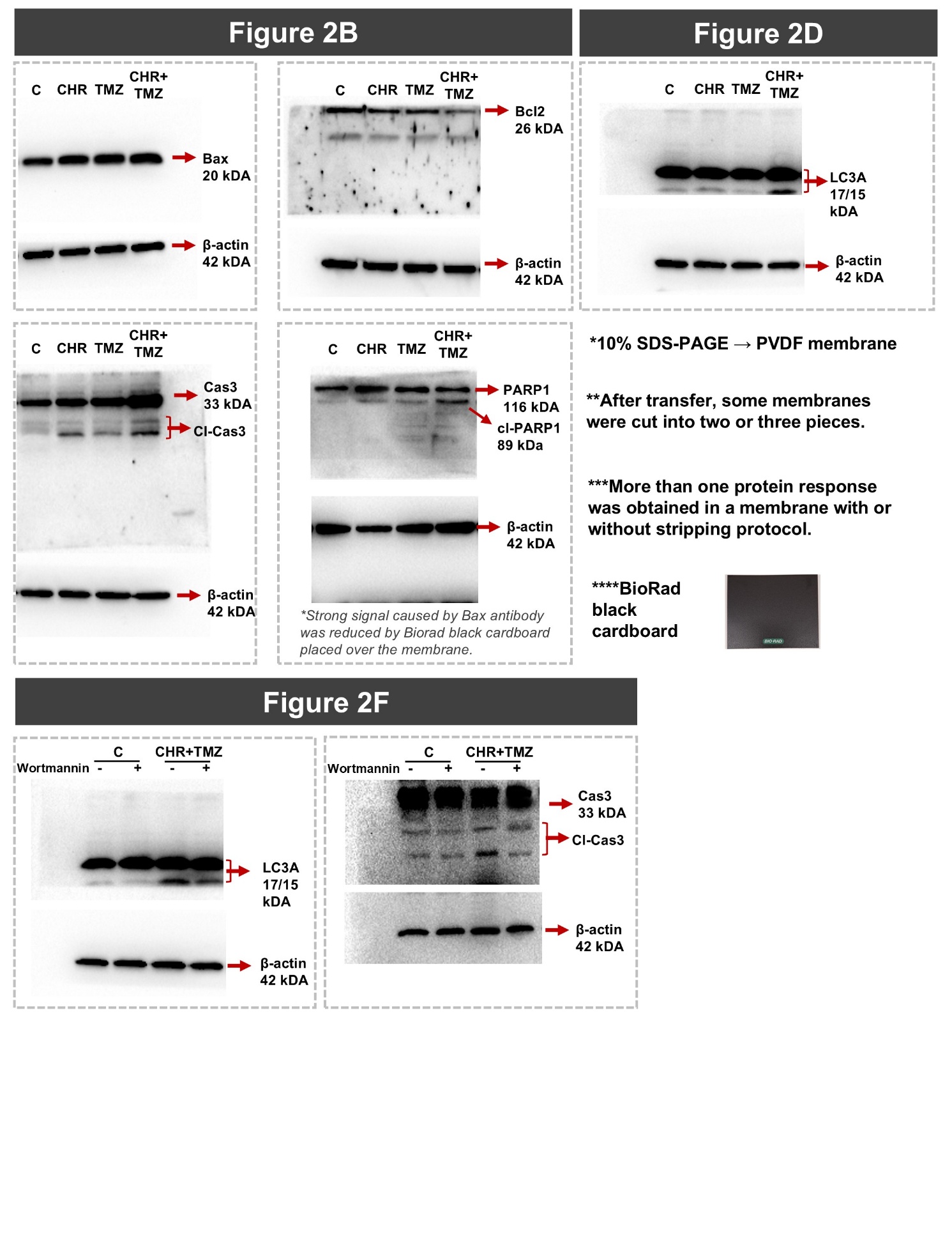


**Figure S2:** Uncropped images of blots and all reference loading controls in Fig.3A-top panel.


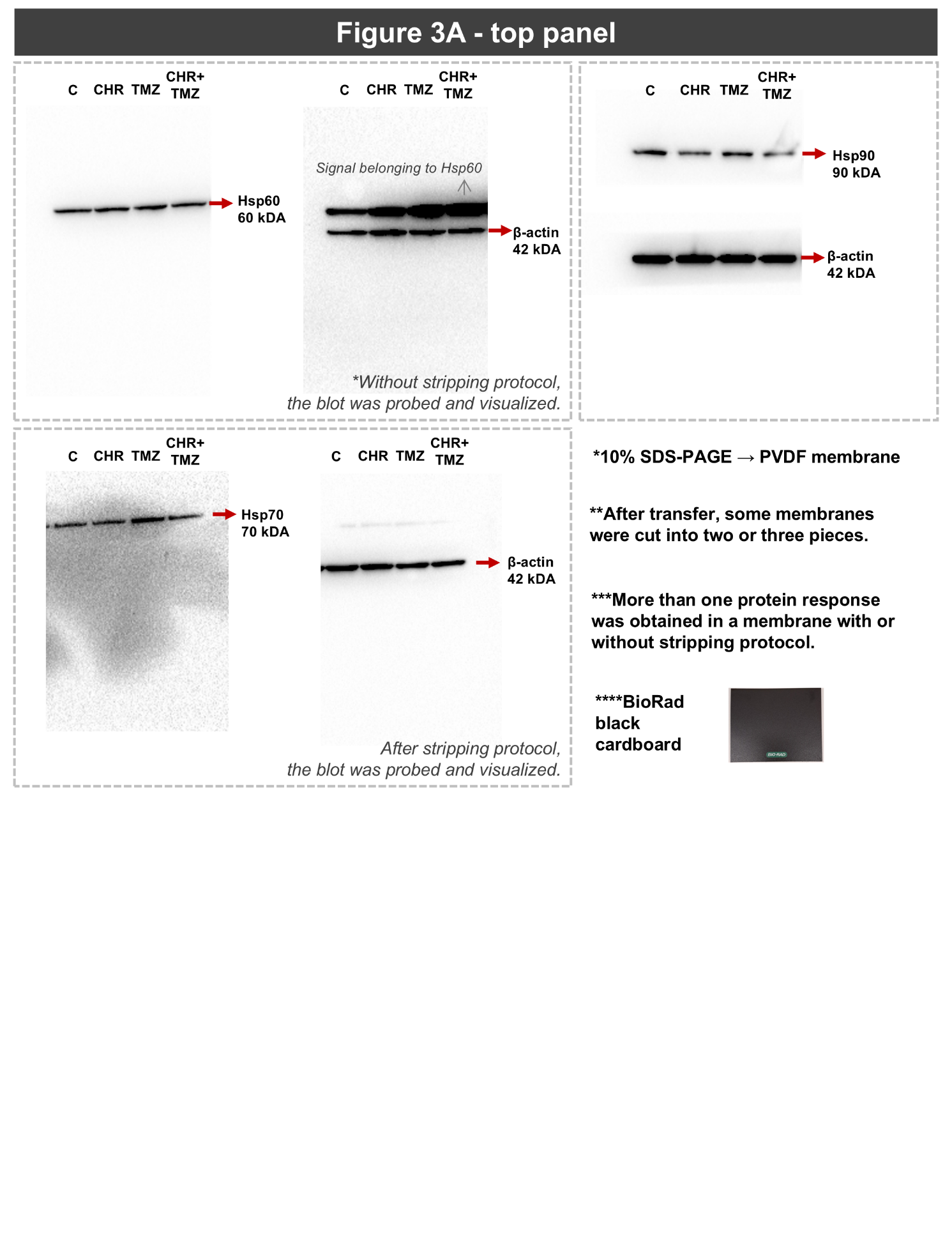


**Figure S3:** Uncropped images of blots and all reference loading controls in Fig.3A-bottom panel.


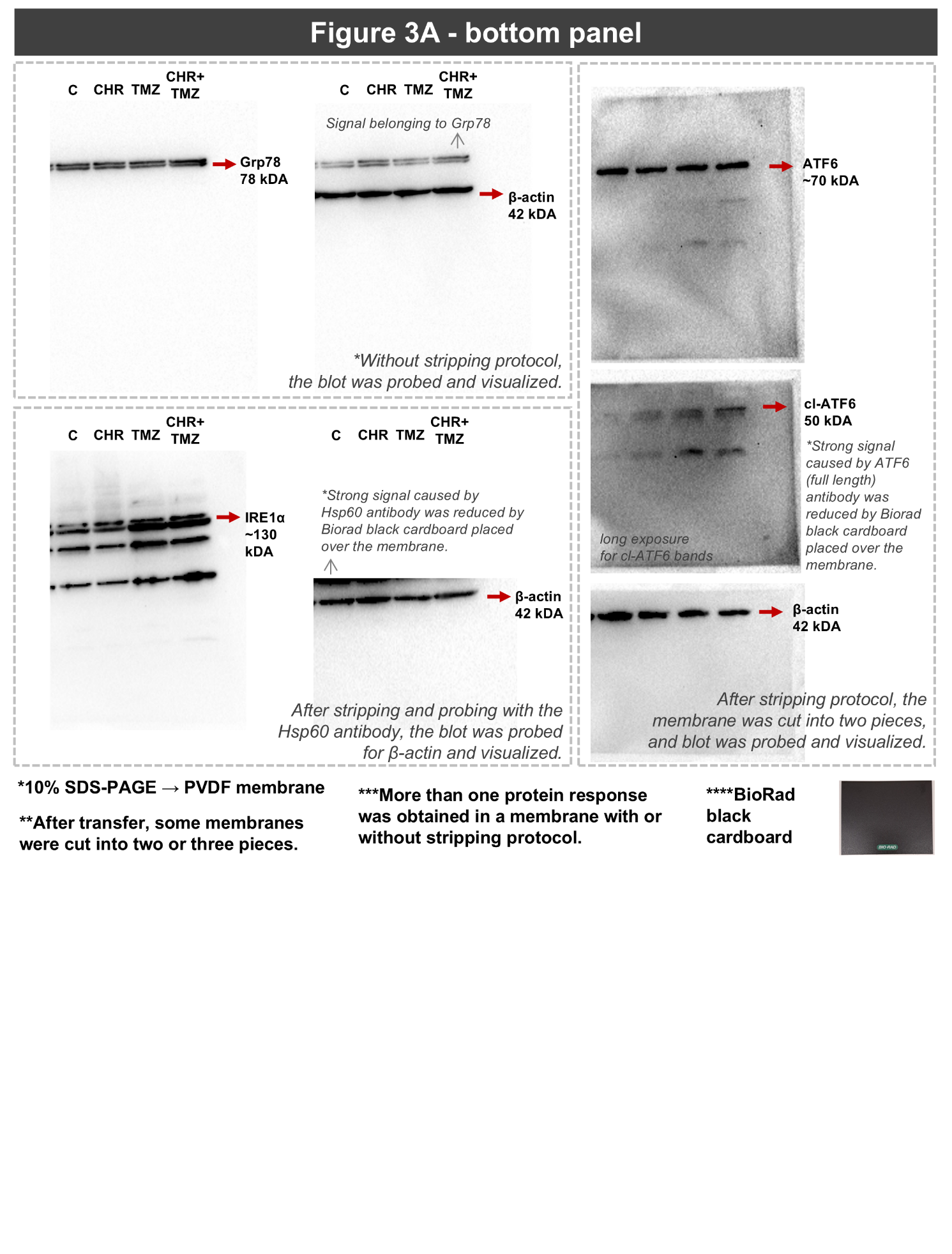


**Figure S4:** Uncropped images of blots and all reference loading controls in Fig.3B and Fig.3D.


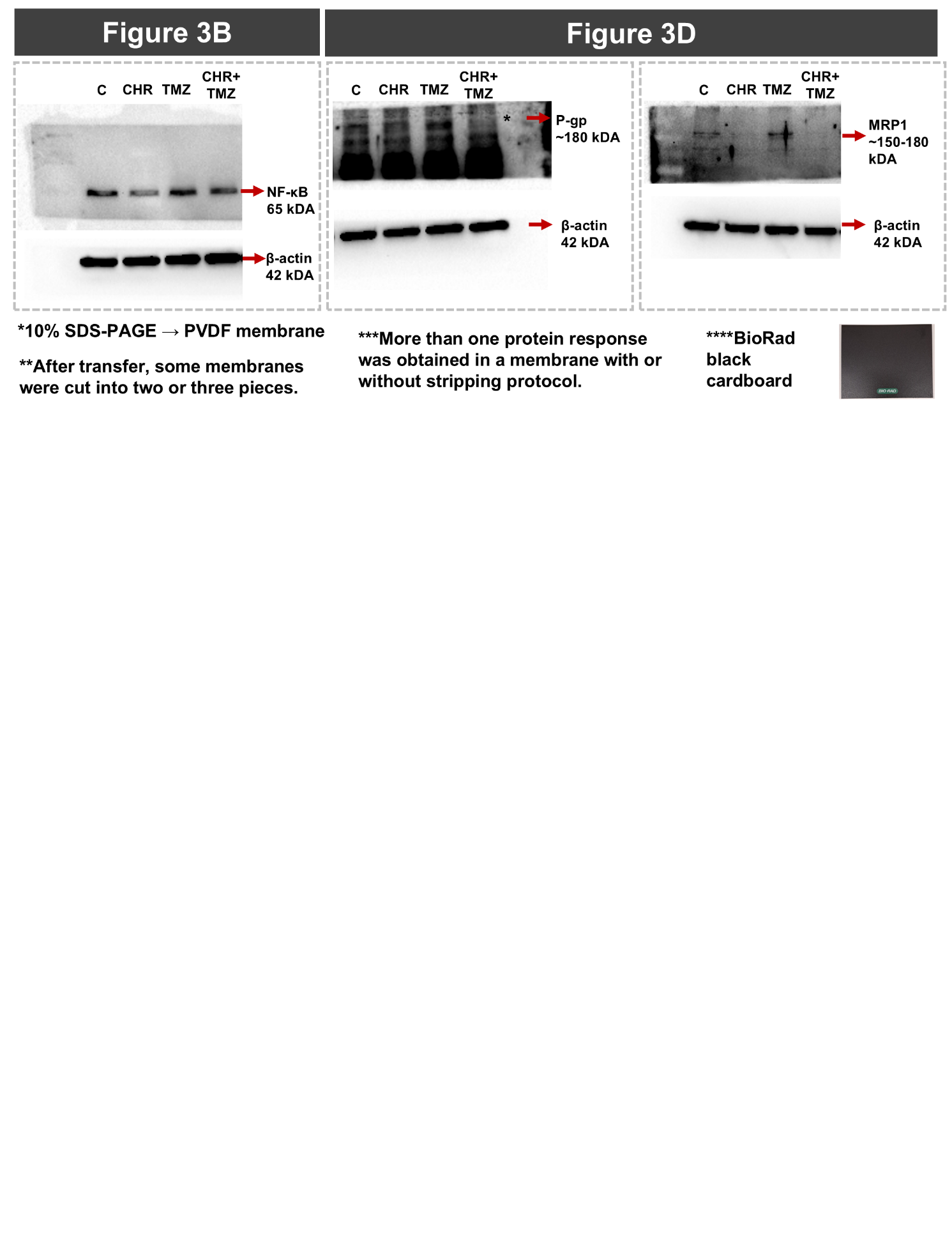


**Figure S5:** Uncropped images of blots and all reference loading controls in Fig.4D and Fig.4E.


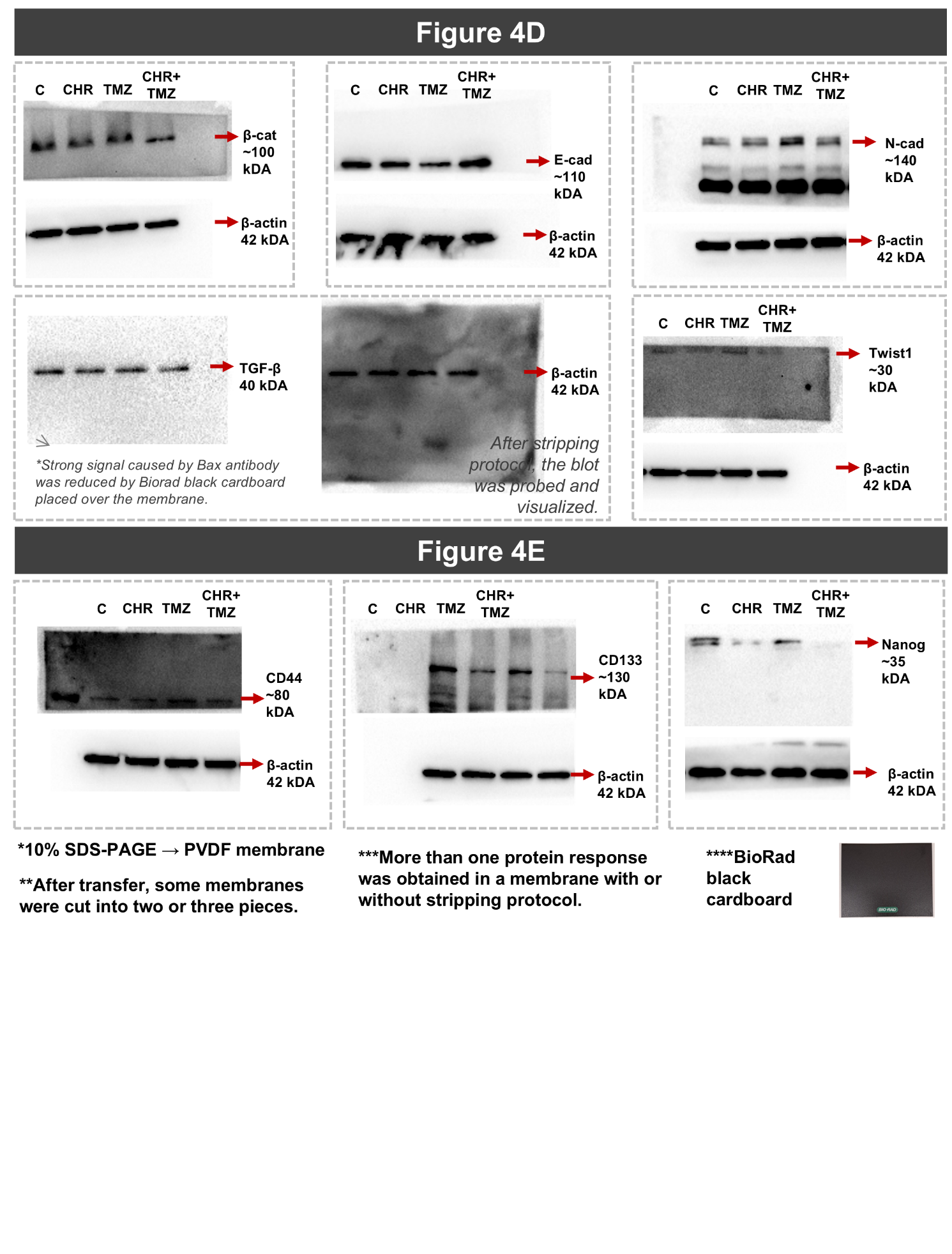

Supplement: Supplementary file 1 [file DataSheet1.docx]
